# A multi-factorial analysis of bone morphology and fracture strength of rat femur in response to ovariectomy

**DOI:** 10.1186/s13018-018-1018-4

**Published:** 2018-12-13

**Authors:** Juan Marcelo Rosales Rocabado, Masaru Kaku, Kosuke Nozaki, Takako Ida, Megumi Kitami, Yujin Aoyagi, Katsumi Uoshima

**Affiliations:** 1 Division of Bio-Prosthodontics, Niigata University Graduate School of Medical and Dental Sciences, Niigata, Japan; 2 Department of Biofunction Research, Institute of Biomaterials and Bioengineering, Tokyo Medical and Dental University, Tokyo, Japan

**Keywords:** Bone, Osteoporosis, Bone quality, Bone architecture, Tissue mineral density, Cortical porosity, Bone fracture risk

## Abstract

**Background:**

Postmenopausal osteoporosis develops due to a deficiency of estrogen that causes a decrease in bone mass and changes in the macro- and micro-architectural structure of the bone, leading to the loss of mechanical strength and an increased risk of fracture. Although the assessment of bone mineral density (BMD) has been widely used as a gold standard for diagnostic screening of bone fracture risks, it accounts for only a part of the variation in bone fragility; thus, it is necessary to consider other determinants of bone strength. Therefore, we aimed to comprehensively evaluate the architectural changes of the bone that influence bone fracture strength, together with the different sensitivities of cortical and trabecular bone in response to ovariectomy (OVX).

**Methods:**

Bone morphology parameters were separately analyzed both in cortical and in trabecular bones, at distal-metaphysis, and mid-diaphysis of OVX rat femurs. Three-point bending test was performed at mid-diaphysis of the femurs. Correlation of OVX-induced changes of morphological parameters with breaking force was analyzed using Pearson’s correlation coefficient.

**Results:**

OVX resulted in a decline in the bone volume of distal-metaphysis trabecular bone, but an increase in distal-metaphysis and mid-diaphysis cortical bone volume. Tissue mineral density (TMD) remained unchanged in both the trabecular and cortical bone of the distal metaphysis but decreased in cortical bone of the mid-diaphysis. The OVX significantly increased the breaking force at mid-diaphysis of the femurs.

**Conclusions:**

OVX decreased the trabecular bone volume of the distal-metaphysis and increased the cortical bone volume of the distal-metaphysis and mid-diaphysis. Despite the reduction in TMD and increased cortical porosity, bone fracture strength increased in the mid-diaphysis after OVX. These results indicate that analyzing a single factor, i.e., BMD, is not sufficient to predict the absolute fracture risk of the bone, as OVX-induced bone response vary, depending on the bone type and location. Our results strongly support the necessity of analyzing bone micro-architecture and site specificity to clarify the true etiology of osteoporosis in a clinical setting.

## Background

The bone is a complex, dynamic, and hierarchical tissue that has various functions, including the architectural framework component of the body, protection of internal organs, and contribution to the maintenance of mineral homeostasis of body fluids. The macro- and micro-architecture of the bone changes with age, pathological conditions, and anatomical location; therefore, bone mechanical properties will change accordingly [1]. Morphologically, there are two distinct types of bone: cortical bone and trabecular bone. The trabecular bone is mainly responsible for rapid bone turnover and regulating systemic mineral homeostasis, while cortical bone is responsible for constructing the body framework [2].

Osteoporosis is a skeletal disease with various pathogenesis and is caused by an imbalance in bone remodeling. Postmenopausal osteoporosis is developed by a deficiency of estrogen, resulting in a decrease in bone mass and changes in the macro- and micro-architectural structure of bone. As a result, postmenopausal osteoporosis leads to the loss of mechanical strength of the bone and increases the bone fracture risk [3]. Clinically, bone mineral density (BMD) measured by dual-energy X-ray absorptiometry (DXA) remains the standard assessment of osteoporosis and bone fracture risk; a *T* score of less than or equal to − 2.5 decrease in BMD determines the onset of the disease [4]. However, BMD is a surrogate marker of bone strength [5], accounting for only 60% of variation in bone fragility [4]. From a mechanical perspective, bones are composed of different hierarchy levels, namely, whole bone geometry, microstructural properties, and intrinsic material properties [6]. BMD is only one factor affecting the material properties of the bone; therefore, other determinants, such as bone mass and bone quality, also need to be considered as potential factors that contribute to bone fracture risks [7]. Bone quality includes several aspects of bone structure and composition, including bone turnover, micro-architecture, the degree and distribution of mineralization, the extent of micro-damage, and the composition of bone matrix and mineral [8–10].

One of the parameters of bone quality, cortical porosity, occurs as a result of accelerated remodeling at the inner surface of long bones and causes trabeculation of the cortical bone. Recent studies revealed that cortical porosity also affects the mechanical properties of the bone [11, 12]. Accelerated bone resorption at the inner side of the cortical bone is frequently observed in patients with osteoporosis, and persistence of this condition leads to intra-cortical porosity, which can compromise bone strength [8]. Studies in humans have shown a consistent relationship between cortical bone porosity and bone fracture risk which, notably, was largely undetected by conventional DXA-based BMD measurements [9, 13]. Currently, the evaluation of the parameters of bone quality has not been adopted for general diagnostic purposes. The contribution of each parameter of bone quality towards the risks of fracture is still unclear. Moreover, the limited resolution of radiographic imaging does not permit the detection of detailed architectural changes in the bone.

Development of site-specific osteoporosis in the trabecular bone is one of the most reproducible biological responses in ovariectomy (OVX) animal models; therefore, OVX has been widely used to understand the pathophysiology of osteoporosis and to evaluate the efficacy of anti-bone resorption treatment [14, 15]. On the contrary, the non-uniform bone reaction in response to OVX complicates the evaluation and understanding of osteoporosis pathogenesis [16–18]. It has been reported that OVX-induced osteoporosis caused reduction of the trabecular bone volume at the proximal tibial metaphysis, but exhibited no marked change in the cortical bone volume at the mid-diaphysis of the tibia and femur in rats [19]. Although cortical bone, the major constituent of skeletal bone, is more responsible for bone strength, its response to OVX is still controversial [20].

While many studies have been conducted for decades, non-uniform skeletal response to OVX, especially between cortical bone and trabecular bone, tend to be overlooked. Moreover, given the importance of bone micro-architecture as a volumetric structure in the evaluation of fracture risk, it is necessary to consider other determinants of bone strength [8, 9]. Therefore, in the present study, through the use of high-resolution computed tomography and data-analyzing software to obtain 3D reconstructions, we aimed to comprehensively evaluate the architectural changes of the bone that influence bone fracture strength, together with different sensitivities of the cortical and trabecular bones in response to OVX.

## Methods

### Experimental animals and surgical procedures

**Fig. 1:**
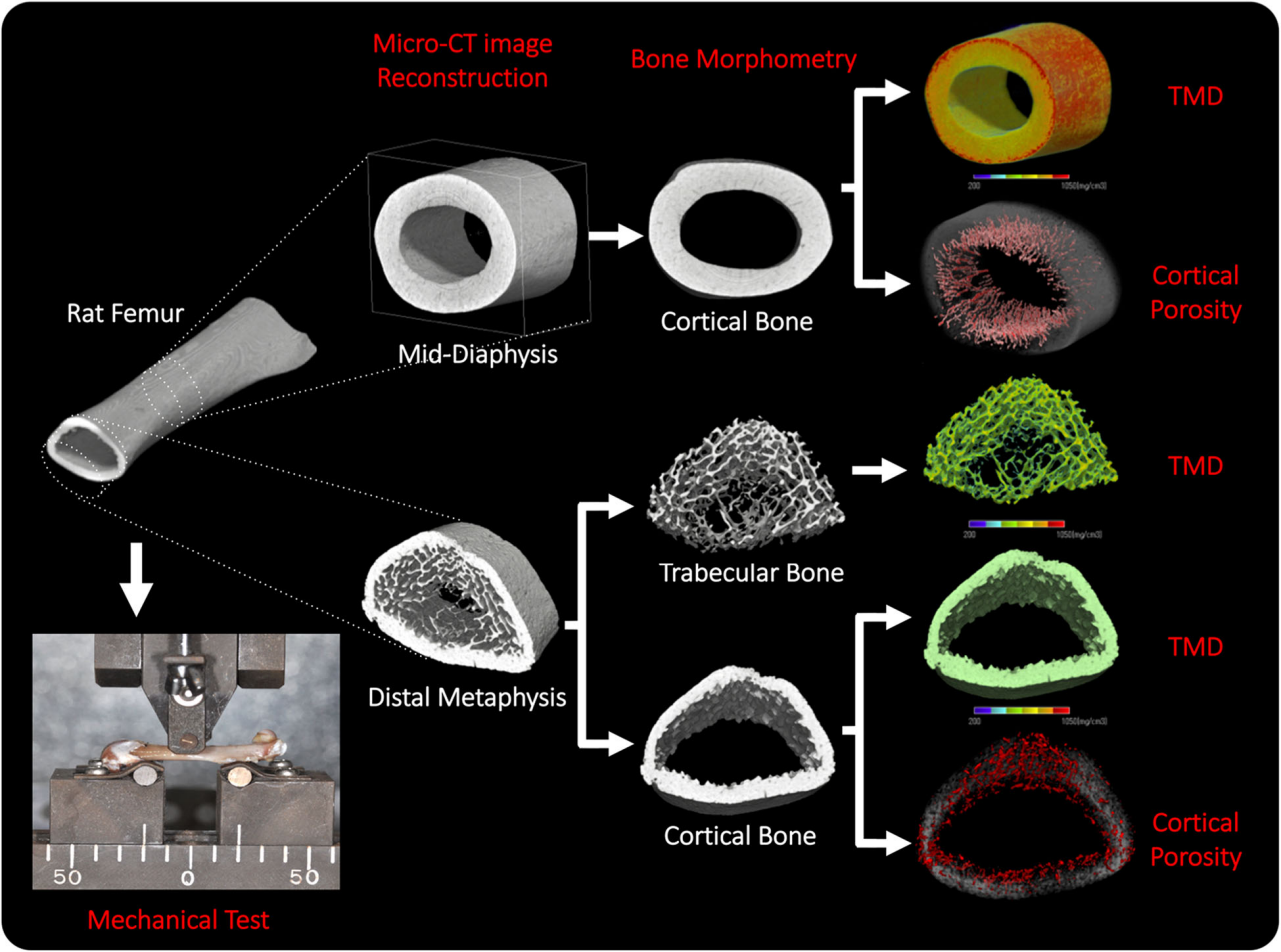
Experimental workflow of the study. Bone morphometry and a mechanical test were performed on the same sample. First, X-ray micro-computed tomography was undergone and three-dimensional reconstruction of the mid-diaphysis and distal metaphysis regions of interest was done. Trabecular bone and cortical bone were separately analyzed at the distal metaphysis. After reconstruction, morphological, tissue mineral density (TMD), and cortical porosity analyses were calculated. After acquiring images, samples were subjected to a three-point mechanical test

A total of 13 female Wistar rats were purchased from Charles River Japan (Yokohama, Japan). Rats were randomly divided into two groups: six rats in the control group and seven rats in the ovariectomy-induced osteoporosis group (OVX group). Bilateral ovariectomy [21, 22] was performed in the OVX group at 10 weeks of age according to the protocol and the ethical guidelines of Niigata University (36-9). The animals were provided with commercially available pellet food and water ad libitum and kept under controlled light conditions of 12/12-h light/dark cycles throughout the experiment. Eight weeks after OVX was performed, all animals were euthanized, and both femurs were excised and immediately fixated in 4% formaldehyde for 3 days at 4 °C. Bone morphometry, cortical porosity, and mechanical testing were performed on the same sample. The experimental workflow of this study is shown in Fig. 1.

### X-ray micro-computed tomography imaging

Micro-computed tomography (micro-CT) images of a section (3.5 mm × 4.5 mm × 2.0 mm) of the femur’s mid-diaphysis were taken. Scanning conditions of 75 Kv, 10 μA, and 9 μm of thickness were applied to obtain a detailed image of the sample, using ELE-scan micro-tomography (Nittetsu Elex Co. Ltd., Tokyo, Japan). A section of the distal metaphysis of the femur was also scanned to analyze the rat femur trabecular bone. The settings for this portion were similar to the mid-diaphysis at 75 Kv and 10 μA; however, since the area to be scanned was wider than the mid-diaphysis, the thickness of each slide was set to 14 μm. Along with the samples, a commercially available BMD phantom (Ratoc Systems, Tokyo, Japan) with different known densities was scanned using the same scan setting.

### Three-dimensional reconstruction and bone morphometry

Three-dimensional (3D) volume reconstructions were done with the ELE-scan software and converted into stacks of TIFF files. Based on the image stacks, a 3D model of the samples was reconstructed and further analysis was performed using Tri/3D-BON software (Ratoc Systems, Tokyo, Japan) [23]. For the analysis of the femur cortical bone, a region of interest (ROI) of 3.5 mm × 4.5 mm × 2.0 mm in size was taken from a section of the mid-diaphysis area. Trabecular bone was analyzed using a 5.5 mm × 7.0 mm × 1.75 mm section of the distal metaphysis. Analysis parameters used complied with the guidelines for the assessment of bone microstructure in rodents using micro-CT [24].

### Cortical porosity and tissue mineral density analysis

Mid-diaphysis cortical porosity analysis was performed using an intensity threshold of 170 for all samples. Measurements were performed according to the protocols and manual guidelines provided by the software company. TMD analysis was performed using the BMD phantom as a density reference for all samples.

### Three-point bending test

Following micro-CT imaging, samples were analyzed by a three-point universal testing machine (EZ Graph, Shimadzu, Kyoto, Japan). Three-point bending strength was set with a length span of 20 mm between the support points. The press head as well as the two support points were round to avoid shear load and damage to the samples [25]. Samples were positioned horizontally, with the anterior surface facing upwards while the compressive force was directed perpendicular to the center of the bone shaft. Each bone was loaded with a constant crosshead speed of 1 mm/min until mechanical failure was reached. Data were obtained from the universal testing machine using Trapezium X v1.1.5 software (EZ Graph, Shimadzu, Tokyo, Japan). Data were then converted into a load-displacement plot, where the load is the force applied in newtons (N) and the displacement is in millimeters (mm). In order to determine the intrinsic properties of a material, the load-displacement data were further converted into a stress-strain graph [26], and mechanical properties were calculated.

### Statistical analysis

All data were processed and analyzed using the independent Student’s *t* test analysis of variance to find the statistical difference between groups. A *P* value less than 0.05 was considered statistically significant. The correlations of regression models were evaluated using Pearson’s determination coefficient *R*^2^ to explore the relationship between the morphological characteristics and biomechanical parameters [27, 28].

## Results

### Body weight

**Fig. 2:**
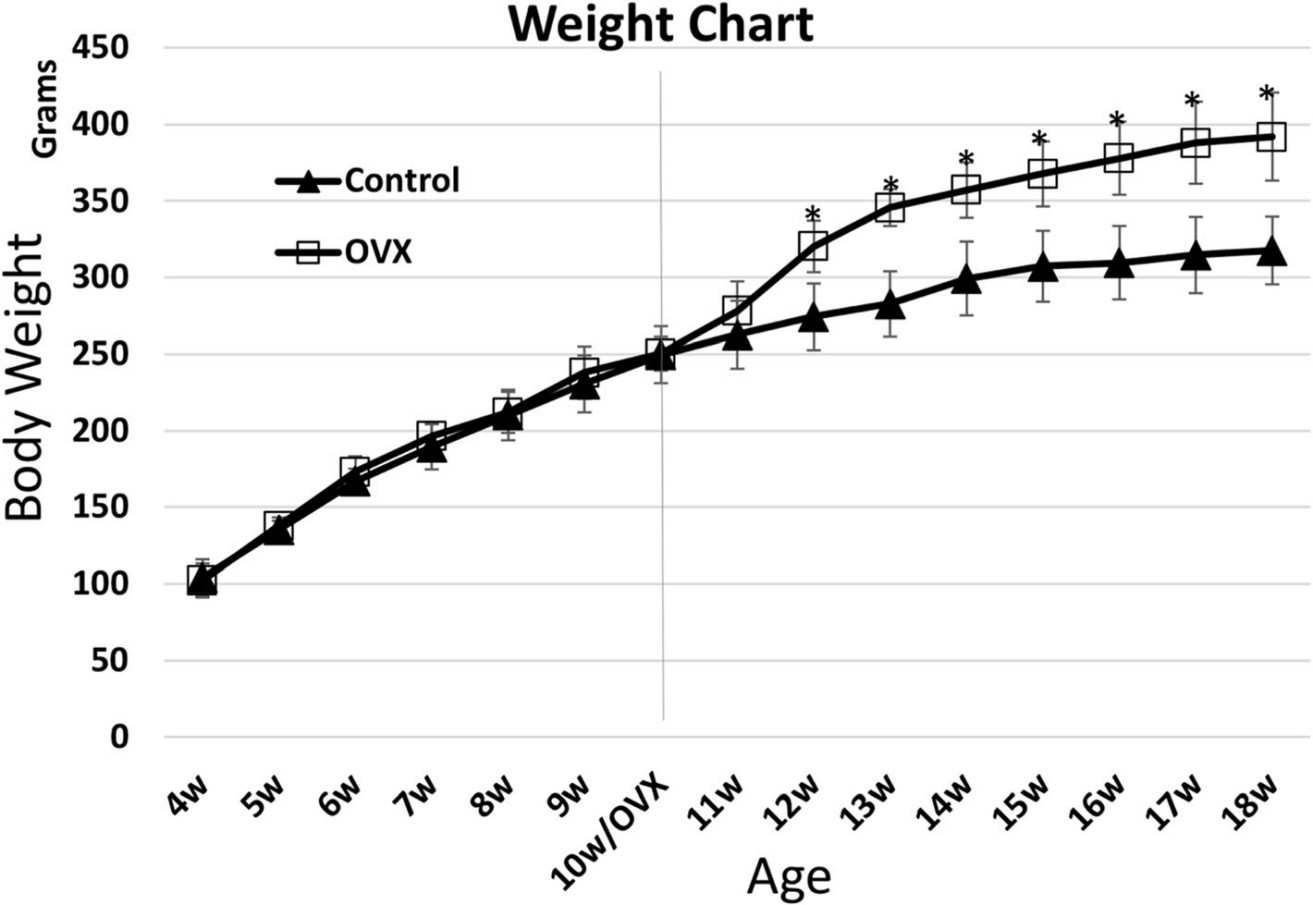
Body weight changes in experimental rats. Ovariectomy (OVX) was performed at 10 weeks of age. Significant weight change became evident 2 weeks after OVX, and the difference persisted throughout the rest of the experimental period. *P < 0.05

Weight changes in the experimental animals were recorded throughout the experimental period (Fig. 2). During the period before OVX, both groups showed a steady increase in body weight, without any significant difference between groups. Two weeks after the bilateral OVX operation, the OVX group showed a significant increase in body weight compared to that in the control group and maintained this increase throughout the rest of the experimental period (up to 18 weeks).

### Morphological characteristics of the femur distal-metaphysis

**Fig. 3:**
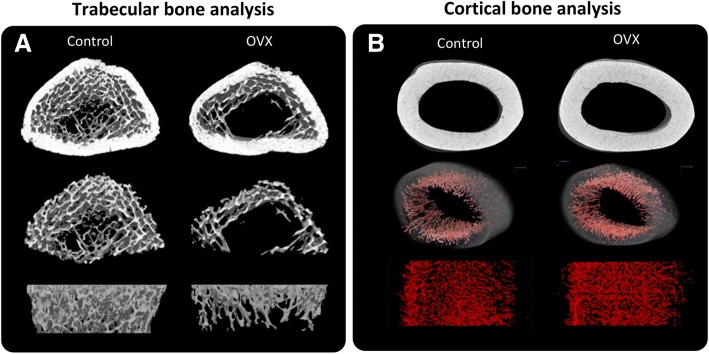
Trabecular bone and cortical bone analysis. **a** Representative reconstructed micro-CT images of the femur distal metaphysis. Top, cortical and trabecular bone combined. Middle, extraction of the trabecular portion. Bottom, lateral view of the extracted trabecular portion. The OVX group showed a marked reduction in trabecular bone. **b** Representative reconstructed micro-CT images of the femur mid-diaphysis. Top, the OVX group showed an increased diameter of the femur mid-diaphysis and negligible amount of trabecular bone exist. Middle, reconstructed images of cortical porosity (red) within the cortical bone. Bottom, lateral view of the reconstructed cortical porosity extracted from bone

Representative 3D-reconstructed images of femur distal-metaphysis are shown in Fig. 3a. The numerical results of the trabecular bone analysis at femur distal-metaphysis are shown in Table 1. Eight weeks after OVX, while total volume (TV) was unchanged, a significant decrease was observed in bone volume (BV), bone surface (BS), bone surface density (BS/TV), and bone volume fraction (BV/TV). Meanwhile, trabecular separation (Tb.Sp), trabecular space (Tb.Spac), and trabecular thickness (Tb.Th) increased while trabecular number (Tb.N) decreased in the OVX group.

**Table 1:** Trabecular morphological characteristics of the distal metaphysis

Trabecular bone analysis of femur distal metaphysis.								
Parameter	Abbreviation	[unit]	Control		OVX		*t* test	
			Mean	± SD	Mean	± SD	*P*	
Bone morphology								
Total volume	TV	[mm^3^]	13.629	1.170	14.111	0.542	0.386	
Bone volume	BV	[mm^3^]	2.363	0.494	0.940	0.294	< 0.001	*
Bone surface	BS	[mm^2^]	85.559	13.695	35.572	9.290	< 0.001	*
Bone surface density	BS/TV	[mm^2^/mm^3^]	6.304	1.121	2.509	0.575	< 0.001	*
Bone volume fraction	BV/TV	[%]	17.429	4.070	6.622	1.849	0.001	*
Trabecular separation	Tb.Sp	[um]	270.266	56.679	775.873	163.517	< 0.001	*
Trabecular space	Tb.Spac	[um]	325.141	53.808	828.254	161.481	< 0.001	*
Trabecular thickness	Tb.Th	[um]	84.323	3.004	90.693	1.999	0.002	*
Trabecular number	Tb.N	[1/mm]	1.502	0.336	0.606	0.151	0.001	*
Trabecular width	Tb.W	[um]	123.125	5.832	121.354	4.196	0.551	
Bone mineral analysis								
Bone mineral content	BMC	[mg]	1.406	0.351	0.506	0.099	0.001	*
Volumetric bone density	vBMD (BMC/TV)	[mg/cm^3^]	102.467	25.289	39.414	11.199	0.001	*
Tissue mineral density	TMD (BMC/BV)	[mg/cm^3^]	605.150	47.774	599.283	17.545	0.787	

*P < 0.05

For mineral content, we analyzed TMD instead of BMD. TMD differs from BMD; TMD is calculated from the average attenuation value of the bone tissue only, whereas BMD assessment will include attenuation values from non-bone soft tissue in addition to the bone tissue. In trabecular bone, TMD was not statistically different between the control and OVX groups. Bone mineral content (BMC) and volumetric bone mineral density (vBMD) significantly decreased in the OVX group, reflecting the reduction of bone volume (BV).

The numerical results of the cortical bone analysis at the femur distal-metaphysis are shown in Table 2. The OVX group had a significant increase in total volume (TV), cortical bone volume (Ct.V), total cross-sectional area (Tt.Ar), cortical bone area (Ct.Ar), periosteal perimeter (Ps.Pm), and cortical thickness (Ct.Th). Although cortical area fraction (Ct.Ar/Tt.Ar), center-line length (CLL), total pore volume (Po.V), and cortical porosity (Ct.Po) were not significantly different, they had a tendency to be increased in the OVX group. Bone mineral content (BMC) increased in the OVX group due to the increases in cortical bone volume (Ct.V). TMD between the control and OVX groups was not significantly different.

**Table 2:** Cortical morphological characteristics of the distal metaphysis

Cortical bone analysis of femur distal metaphysis.								
Parameter	Abbreviation	[unit]	Control		OVX		*t* test	
			Mean	± SD	Mean	± SD	*P*	
Bone morphology								
Total volume	TV	[mm^3^]	26.976	1.075	28.785	1.005	0.010	*
Cortical bone volume	Ct.V	[mm^3^]	12.257	0.534	13.481	0.643	0.003	*
Total cross-sectional area	Tt.Ar	[mm^2^]	15.292	0.610	16.318	0.570	0.010	*
Cortical bone area	Ct.Ar	[mm^2^]	6.948	0.303	7.642	0.364	0.003	*
Cortical area fraction	Ct.Ar/Tt.Ar	[%]	45.327	2.142	46.827	1.346	0.148	
Periosteal perimeter	Ps.Pm	[um]	14,807.643	218.362	15,230.109	338.133	0.021	*
Center line length	CLL	[um]	13,397.415	318.452	13,566.205	297.338	0.348	
Cortical thickness	Ct.Th	[um]	526.738	17.219	578.632	35.623	0.008	*
Total pore volume	Po.V	[mm^3^]	0.127	0.026	0.190	0.084	0.099	
Cortical porosity	Ct.Po	[%]	1.019	0.192	1.371	0.552	0.155	
Bone mineral analysis								
Bone mineral content	BMC	[mg]	10.663	0.438	11.828	0.743	0.006	*
Tissue mineral density	TMD (BMC/Ct.V)	[mg/cm^3^]	862.800	49.970	864.529	21.839	0.940	

*P < 0.05

### Morphological characteristics of the femur mid-diaphysis

Reconstructed images of the femur mid-diaphysis are shown in Fig. 3b. The mid-diaphysis portion of the femur had a negligible amount of trabecular bone; therefore, quantitative analysis could not be done. The results of the cortical bone analysis are shown in Table 3. Quantitative analysis at the femur mid-diaphysis of the samples showed a significant increase of TV, Ct.V, Tt.Ar, Ct.Ar, Ps.Pm, and CLL; however, Ct.Ar/Tt.Ar and Ct.Th showed no differences. Regarding the porosity, Po.V and Ct.Po showed a significant increase in the OVX group. Although BMC was not altered, TMD decreased due to increases of Ct.V in the OVX group.

**Table 3:** Cortical morphological characteristics of the mid-diaphysis

Cortical bone analysis of femur mid-diaphysis.								
Parameter	Abbreviation	[unit]	Control		OVX		*t* test	
			Mean	± SD	Mean	± SD	*P*	
Bone morphology								
Total volume	TV	[mm^3^]	19.576	0.765	20.818	0.701	0.012	*
Cortical volume	Ct.V	[mm^3^]	12.059	0.186	12.610	0.168	< 0.001	*
Total cross-sectional area	Tt.Ar	[mm^2^]	9.798	0.383	10.419	0.351	0.012	*
Cortical bone area	Ct.Ar	[mm^2^]	6.035	0.093	6.311	0.084	< 0.001	*
Cortical area fraction	Ct.Ar/Tt.Ar	[%]	61.684	2.796	61.134	2.016	0.698	
Periosteal perimeter	Ps.Pm	[mm]	11.395	0.180	11.778	0180	0.003	*
Center line length	CLL	[mm]	9.189	0.263	9.545	0.217	0.026	*
Cortical thickness	Ct.Th	[um]	670.039	29.232	685.791	24.097	0.319	
Total pore volume	Po.V	[mm^3^]	0.196	0.047	0.320	0.041	0.002	*
Cortical porosity	Ct.Po	[%]	1.597	0.358	2.461	0.320	0.003	*
Bone mineral analysis								
Bone mineral content	BMC	[mg]	9.164	0.174	9.315	0.178	0.191	
Tissue mineral density	TMD (BMC/Ct.V)	[mg/cm^3^]	740.540	6.868	718.857	8.689	0.001	*

*P < 0.05

### Mechanical test of the femur mid-diaphysis

The results of the femur mid-diaphysis three-point bending test are shown in Table 4. After 8 weeks, the OVX group had a significant increase in breaking force, breaking displacement, and breaking time; however, stiffness did not show a significant difference. Moreover, in analyzing the stress-strain graph, the OVX group showed a significant increase in fracture strength; however, Young’s modulus, ductility, resilience modulus, and toughness did not show significant differences.

**Table 4:** Three-point bending test

Mechanical analysis of rat femur.							
Parameter	[unit]	Control		OVX		*t* test	
		Mean	± SD	Mean	± SD	*P*	
Force-displacement plot							
Breaking force	[N]	102.917	6.379	112.865	4.815	0.013	*
Breaking displacement	[mm]	0.426	0.035	0.482	0.023	0.020	*
Breaking time	[sec]	25.540	2.088	28.650	1.381	0.026	*
Stiffness	[N/m^2^]	302.062	30.749	299.899	35.000	0.912	
Stress-strain graph							
Fracture strength	[MPa]	16.799	1.041	18.423	0.786	0.013	*
Young’s modulus	[MPa]	987.215	99.849	1031.412	45.857	0.357	
Ductility	[%]	0.005	0.002	0.006	0.004	0.476	
Resilience mod	[%]	0.142	0.012	0.154	0.029	0.369	

*P < 0.05

### Correlation analysis

**Fig. 4:**
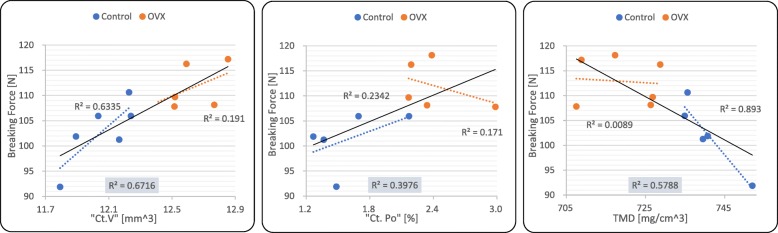
Correlation analysis between the morphological characteristics and breaking force. The correlation lines (black line) with correspondent *R*^2^ values (shaded *R*^2^ box) of the graphs indicate the correlation between two variables within the two groups (control and OVX). Cortical volume (Ct.V) and cortical porosity (Ct.Po) were positively correlated, while tissue mineral density (TMD) showed a negative correlation with the breaking force

To determine the correlation of OVX-induced changes of selected morphological characteristics (i.e., Ct.V, Ct.Po, and TMD) with breaking force, the Pearson correlation coefficient was calculated. The results of the correlation analysis are shown in Fig. 4. A strong positive correlation between Ct.V and breaking force was observed. In contrast, Ct.Po showed a weak positive correlation with breaking force. Conversely, TMD showed a strong negative correlation with breaking force.

## Discussion

The OVX animal model has been widely used to understand the pathophysiology of osteoporosis and to evaluate the efficacy of anti-bone resorption treatment. The OVX-induced early bone loss occurs mainly in the trabecular bone; however, micro-architectural changes in the cortical bone and their correlation with the changes in the trabecular bone are still unclear. Therefore, in the present study, bone morphological parameters were separately analyzed both at cortical and trabecular bones in response to OVX using rat femurs. Interestingly, OVX resulted in contradictory results between trabecular and cortical bone. Bone volume decreased in the trabecular bone of distal metaphysis but increased in the cortical bone of distal metaphysis and mid-diaphysis; TMD remained unchanged in both trabecular and cortical bone of the distal metaphysis but decreased in the cortical bone of the mid-diaphysis. More surprisingly, the fracture strength of mid-diaphysis was significantly increased in response to OVX. The data obtained in this study are summarized in Table 5.

**Table 5:** OVX has different effects on the cortical and trabecular bone in rat femur

Location	Bone type	Bone volume	TMD	Cortical porosity	Breaking force
Distal metaphysis	Trabecular	↓	→	–	–
Distal metaphysis	Cortical	↑	→	→	–
Mid-diaphysis	Cortical	↑	↓	↑	↑

In response to OVX, a significant increase in bone volume and size was observed in the cortical bone of femur metaphysis, which demonstrated a strong positive correlation with the changes in breaking force (Table 4). Increases in the external diameter of the long bones with a concomitant increase in bone strength following OVX in rats have been also previously reported [4, 5, 29–32]. It is important to note that OVX cannot be considered to induce a bone-specific estrogen deficiency. OVX is reported to induce hyperphagia, leading to weight gain and increased adiposity [33], because estrogen regulates food intake via anorexigenic pathways of the central nervous system [34]. In addition, bone-specific deletion of estrogen signaling, using genetically-manipulable transgenic mice, did not show significant phenotypes in cortical bone [35, 36] and phenotypes vary among age, sex, and mouse lines [37]. Further studies are necessary to elucidate the precise function of estrogen-signaling on cortical bone homeostasis.

BMD measurement by DXA has been the gold standard assessment of osteoporosis; however, it accounts only partially towards fracture risk [4]. Although BMD is calculated as the mass of mineral per volume of bone, the images obtained by DXA cannot differentiate the trabecular bone from soft tissue. Due to the limitation of image resolution, the porous trabecular bone is considered as a solid material. Consequently, conventional BMD measurement represents the aspects of both volume and material mineral density of the trabecular bone. Since our data were 3D-based, we calculated the volumetric BMD (vBMD) instead of conventional 2D-based BMD. Our results clearly demonstrated that OVX significantly decreased the volume of trabecular bone at the distal metaphysis, and vBMD decreased accordingly. However, TMD, representing solid material mineral density, did not change. Our high-resolution image analysis revealed that the decline of vBMD by OVX is mainly because of the decreased trabecular bone volume, and not because of the changes in bone mineral density. In addition, OVX decreased the TMD at the cortical bone of the mid-diaphysis, but did not affect the trabecular or cortical bone of the distal metaphysis, indicating a site-specific bone reaction in response to OVX. These results strongly support the necessity of analyzing bone micro-architecture and site specificity to clarify the true etiology of osteoporosis in a clinical setting.

In this study, bone volume, external perimeter, and cortical porosity increased, while TMD decreased in the cortical bone of the femur mid-diaphysis. Increases in cortical thickness and perimeter are associated with higher bone strength. Cortical perimeter and cross-sectional area are crucial geometric parameters of bone strength, because increasing a hollow cylinder diameter provides exponential increases in resistance to bending and torsion without requiring increases in bone mass [8]. In line with our observations, a previous study reported that OVX significantly decreased BMD, but increased cross-sectional area and inertia tolerance on the femoral neck; consequently, femoral necks in OVX rats had greater stiffness than in the controls [29]. A human study also showed that fractures were more frequent in individuals with lower cortical thickness, while no association was seen between fracture and cortical BMD [38], indicating that individuals with identical BMD values can have different architectural characteristics of the cortical bone and, therefore, different fracture risks [9, 13]. It has also been reported that the trabecular connectivity contributes more to the bone mechanical strength than BMD in human calcaneal bone [39]. Thus, the assessment of multiple factors such as bone morphology, bone composition, and bone properties besides BMD is necessary to determine the absolute fracture risk of bone [40].

We observed a contradictory result between the trabecular and cortical bones at the distal metaphysis, which demonstrated increased cortical bone volume but decreased trabecular bone volume. Different sensitivity to OVX between cortical and trabecular bone is most likely due to the available bone surface [41]. In addition, recent studies have made progress elucidating a molecular cue controlling the bone type-specific response to OVX, which involves a differential expression pattern of two distinct estrogen receptors (ER), ERα and ERβ. The predominant form of ER, ERα, is expressed in both cortical and trabecular bone, while ERβ, which acts as a natural antagonist to ERα [35], is highly expressed in trabecular bone compared with that in cortical bone [42]. The different expression patterns of these ERs would lead to different sensitivities to the OVX-induced estrogen deficiency. Nevertheless, more precise analysis and longer periods of observation are needed in order to clearly verify this notion.

In our results, cortical porosity of the mid-diaphysis was significantly higher in OVX rats, but did not have a substantial impact on bone strength. However, it has reported that the increase in cortical porosity associated with the initial stages of the bone remodeling process may actually weaken the bone tissue [43]. In our experimental setting, OVX affected not only cortical porosity, but also other bone morphological parameters, e.g., bone volume and external perimeter, thus, evaluating the absolute contribution of cortical porosity to the fracture strength is particularly challenging. Further studies, such as finite element analysis, will reveal the absolute significance of cortical porosity on the mechanical properties of bone.

## Conclusions

In the present study, OVX resulted in a decline in the bone volume of distal metaphysis trabecular bone, but an increase in the bone volume of the distal metaphysis and mid-diaphysis cortical bone. Despite the reduction in TMD and increases in cortical porosity, bone fracture strength increased in the mid-diaphysis after OVX. This suggests that OVX-induced changes in volume/size seemed to be a major determinant of breaking force at the mid-diaphysis of a rat femur. These results clearly indicate that analyzing a single factor, i.e., BMD, is not sufficient to predict the absolute fracture risk of the bone, as OVX-induced bone response vary, depending on the bone type and location. Taken together, understanding the different sensitivities to estrogen deficiency with regard to their impact on bone location and bone type is essential. Further studies are necessary to elucidate the relationship between site-specific OVX-induced changes in both macro-and micro-morphological characteristics and the fracture risk of bone.

## Abbreviations

3D: Three-dimensional
BMC: Bone mineral content
BMD: Bone mineral density
BS: Bone surface
BV: Bone volume
CLL: Center line length
Ct.Ar: Cortical bone area
Ct.Po: Cortical porosity
Ct.Th: Cortical thickness
Ct.V: Cortical bone volume
ER: Estrogen receptors
Micro-CT: Micro-computed tomography
OVX: Ovariectomy
Po.V: Total pore volume
Ps.Pm: Periosteal perimeter
ROI: Region of interest
Tb.N: Trabecular number
Tb.Sp: Trabecular separation
Tb.Spac: Trabecular space
Tb.Th: Trabecular thickness
TMD: Tissue mineral density
Tt.Ar: Total cross-sectional area
TV: Total volume
